# Lateral Flow Assay for Hepatitis B Detection: A Review of Current and New Assays

**DOI:** 10.3390/mi14061239

**Published:** 2023-06-12

**Authors:** Norhidayah Abu, Noremylia Mohd Bakhori, Rafidah Hanim Shueb

**Affiliations:** 1 Department of Medical Microbiology and Parasitology, School of Medical Sciences, Universiti Sains Malaysia, Kubang Kerian 16150, Kelantan, Malaysia; 2 Advanced Materials Research Centre (AMREC), SIRIM Berhad, Lot 34, Jalan Hi-Tech 2/3, Kulim Hi-Tech Park, Kulim 09000, Kedah, Malaysia; noremylia@sirim.my

**Keywords:** hepatitis B virus, lateral flow assay, accuracy, serological, molecular

## Abstract

From acute to chronic hepatitis, cirrhosis, and hepatocellular cancer, hepatitis B infection causes a broad spectrum of liver diseases. Molecular and serological tests have been used to diagnose hepatitis B-related illnesses. Due to technology limitations, it is challenging to identify hepatitis B infection cases at an early stage, particularly in a low- and middle-income country with constrained resources. Generally, the gold-standard methods to detect hepatitis B virus (HBV) infection requires dedicated personnel, bulky, expensive equipment and reagents, and long processing times which delay the diagnosis of HBV. Thus, lateral flow assay (LFA), which is inexpensive, straightforward, portable, and operates reliably, has dominated point-of-care diagnostics. LFA consists of four parts: a sample pad where samples are dropped; a conjugate pad where labeled tags and biomarker components are combined; a nitrocellulose membrane with test and control lines for target DNA-probe DNA hybridization or antigen-antibody interaction; and a wicking pad where waste is stored. By modifying the pre-treatment during the sample preparation process or enhancing the signal of the biomarker probes on the membrane pad, the accuracy of the LFA for qualitative and quantitative analysis can be improved. In this review, we assembled the most recent developments in LFA technologies for the progress of hepatitis B infection detection. Prospects for ongoing development in this area are also covered.

## 1. Introduction

Hepatitis refers to inflammation of the liver, as the ancient Greek word hepa means liver and itis means inflammation. The hepatitis B virus (HBV) is a significant contributor to the development of liver cirrhosis, hepatitis, and liver cancer. This virus is often referred to as a “silent epidemic” as many infected individuals, whether newly or chronically infected, do not display any symptoms. The World Health Organization (WHO) reports that over 240 million people globally have chronic HBV infections. Without a concerted and expedited response, it is projected that the number of individuals with this virus will remain at current high levels for the next several decades, resulting in an estimated 20 million deaths between 2015 and 2030 [1]. To combat this issue, the WHO released its first official strategy in October 2015, aimed at reducing the morbidity and mortality associated with chronic HBV and Chronic Hepatitis C Virus (CHC) infections by 2030 [2,3].

Thus, early detection of this HBV infection is crucial in controlling the spread of the disease and saving the world from its negative impacts. The analysis of biomarker levels in bodily fluids establishes the degree of HBV infection [4]. The most used assays for the detection of HBV are serological assays, such as enzyme immunoassay (EIA), also known as enzyme-linked immunosorbent assay (ELISA) [5,6,7,8]; chemiluminescent microparticle immunoassay (CMIA) [9,10,11]; electrochemiluminescence immunoassay (ECLIA) [12,13,14,15,16]; and molecular assays, such as polymerase chain reaction (PCR) [17,18,19,20] and real-time PCR [21,22]. The screening and diagnosis of the laboratory-based serological assay/immunoassays are based on immunoreactions between antigens and antibodies connected to enzymatic reactions in a serological sample, whilst the molecular assay reveals details regarding the existence of unusual deoxyribonucleic acid (DNA) strands, estimation of the infectious risk, selection of a course of treatment, and infection monitoring [23,24,25].

Any treatment process must begin with an accurate and dependable diagnosis of the disease. Particularly in the early stages of infection or when there is a low viral load, several traditional HBV detection techniques, such as ELISA, may not be as sensitive as they claim to be. This can cause the occurrence of false positives or false negative results. False positives and false negatives are critical considerations in HBV detection that may lead to misdiagnosis and delayed interventions. A study on 48 samples from blood donors at the Hospital University Science Malaysia, Malaysia comparing the detection of HBV between PCR and ELISA, showed that PCR recorded 14.58% of the total positive samples compared with 6.25% anti-HBV ELISA positive [5]. Thus, the percentage of false negativity of HBV-ELISA screening compared to the PCR test was 2.4%. Moreover, numerous stages and extended processing times are needed for molecular assays, such as PCR, which might delay the release of test findings. Hence, the assay can be relatively expensive due to the high cost of the reagents, equipment and maintenance. The COBAS^®^ Ampliprep/COBAS^®^ TaqMan^®^ system (Roche Diagnostics, Indianapolis, IN, USA) or the Xpert^®^HBV Viral Load Test (Cepheid, Sunnyvale, CA, USA) are commercial molecular test systems that are used to assess HBV viral load [26]. This may make it more difficult to make quick decisions when managing patients. Thus, the accessibility of the PCR test and real-time PCR tests is low in resource-constrained settings. As a result, research on the development of point-of-care test (POCT) devices has concentrated on enhancing their portability and accuracy. As per the ASSURED (affordable, sensitive, specific, user-friendly, rapid and robust, equipment-free, and deliverable to end-users) [27] criteria developed by the WHO, lateral flow assay (LFA) offers a solution as an analytical method for POCT detection since a result can be obtained in less than 30 min, without the need for trained personnel and specialized equipment [28,29]. The development of LFA will contribute to expanding the screening of HBV especially in low and middle-income countries with limited resources or where immediate results are crucial for timely intervention. This review summarizes the advancement of LFA in HBV detection. We divided this review into three sections: an overview of biomarkers and diagnosis of HBV, structures and types of LFA, and lastly, current and emerging technology in LFA for HBV detection. Additionally, future strategies and approaches based on LFA technology to boost the diagnostic accuracy of HBV infection are discussed.

## 2. Diagnosis of HBV

**Figure 1:**
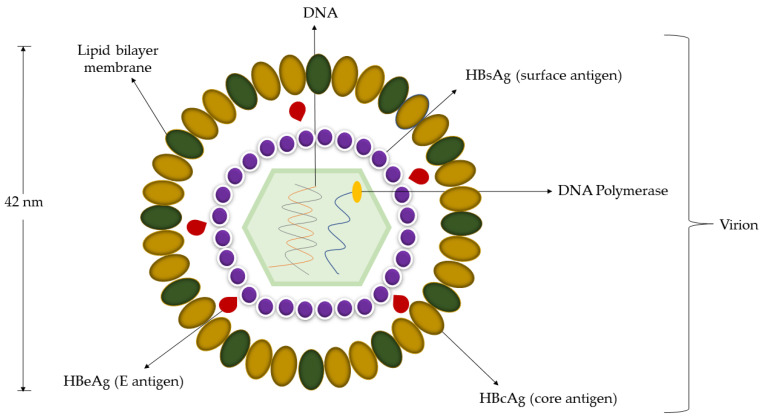
The structure of the hepatitis B virus.

HBV is an enveloped virus that has a partially double-stranded circular DNA genome ranging from 3.0 to 3.4 kilobases (kb) [30,31,32]. The structural size of the HBV virus was first demonstrated in the serum of patients with Australia-antigen-associated hepatitis by Dane et al. when they published the electron microscope images of the particles in 1970 [33]. The particles were reported to be approximately 42 nm in diameter and no morphological changes of particles were observed, indicating the resistance of the serum hepatitis virus. The inner nucleocapsid of the virus is composed of the hepatitis B core antigen (HBcAg) and hepatitis B envelope antigen (HBeAg), functionalized with virally encoded polymerase and partially double-stranded DNA genome. This inner nucleocapsid is surrounded by filamentous hepatitis B surface antigen (HBsAg) particles (Figure 1) [34,35,36].

Serological assay and molecular assay are well-known techniques used to determine HBV infection. The first step in the diagnosis of HBV infection is serological detection of specific viral antigens and antibodies [31,37,38]. The presence of these serological biomarkers will not only determine the infection, but also the clinical phase, either acute or chronic infection, in patients [39]. The biomarkers of HBV consist of hepatitis B surface antigen (HBsAg), hepatitis B surface antibody (HbsAb or Anti-HBs), hepatitis B envelop antigen (HbeAg), hepatitis B envelop antibody (HbeAb), hepatitis B core antigen (HbcAg) and hepatitis B core antibody (HbcAb or Anti-HBc) [30,40]. HBV DNA measurement is a direct indicator of the viral load. It is detected at the earliest stage of infection (one month after HBV exposure), rises to a peak level about 3 months later, and then steadily declines in chronic infection or vanishes at the time of HBV recovery [41]. HbsAg, the first virological marker to be found on the viral surface, indicates an acute HBV infection [42,43,44]. As HbsAg is a predominant viral marker, HBV serology can typically be detected as early as 1–2 weeks or as late as 11–12 weeks after the first infection. Although HbeAg can be detected in most acute phases of infections, it has minimal clinical significance. Loss of serum HbsAg and HBV DNA, as well as the presence of anti-HBs, indicate the resolution of infection and recovery [45,46]. The presence of anti-HBc antibodies is sometimes the only serological sign of HBV infection [47]. Persistence of anti-HBc is a hallmark of chronicity. The presence of HBsAg in the serum for over 6 months also defines the chronicity of the infection [31,48]. The clinical interpretation of HBV biomarkers is summarized in Table 1.

## 3. Principle of LFA

**Figure 2:**
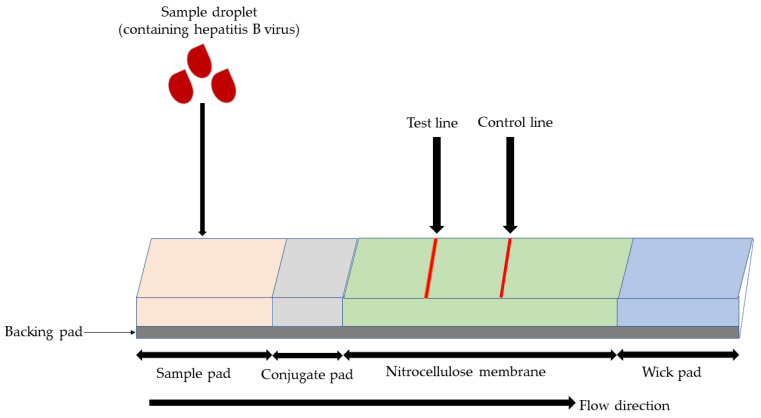
Typical structure of LFA. The assay is composed of a sample pad, conjugate pad, nitrocellulose membrane and wicking pad, fixed on the backing pad.

LFA emerged for the first time at the end of the 1960s, used to monitor serum proteins. The first LFA was produced in 1976 to detect human chorionic gonadotropin (hCG) in urine [49]. The structure of LFA generally consists of several zones constituted by segments of different membrane materials, such as sample pad, conjugated pad, test pad, absorbent pad and backing pad [50,51]. A typical LFA strip consists of membranes that overlap with one another and are mounted on a backing card (Figure 2). LFA is a one-step assay without the need to manually interfere with operation during the whole detection procedure. The principle of this test is based on antibody-antigen-specific interaction [52]. LFA has been widely used in detecting various molecules, such as cancer markers, microorganisms, mycotoxins, heavy metals, and pesticides. The low cost, rapidness and portability of LFA make it a favored alternative tool for the primary screening of infectious diseases and monitoring of chronic diseases [53,54].

Currently, colloidal gold nanoparticles (AuNP) are the most widely used label in commercial LFA systems because of their favorable biocompatibility, ease of production, and potent optical characteristics in the presence of various analytes for capture and detection in liquid and dried forms [55,56]. Numerous studies have been carried out by using LFA-labeled AuNP for various detections, such as African swine fever virus (ASFV) major capsid protein p72 [57], non-structural 1 (NS1) secretory protein of Japanese Encephalitis Virus (JEV) [58], and the nucleocapsid protein of SARS-CoV-2 [59].

**Figure 3:**
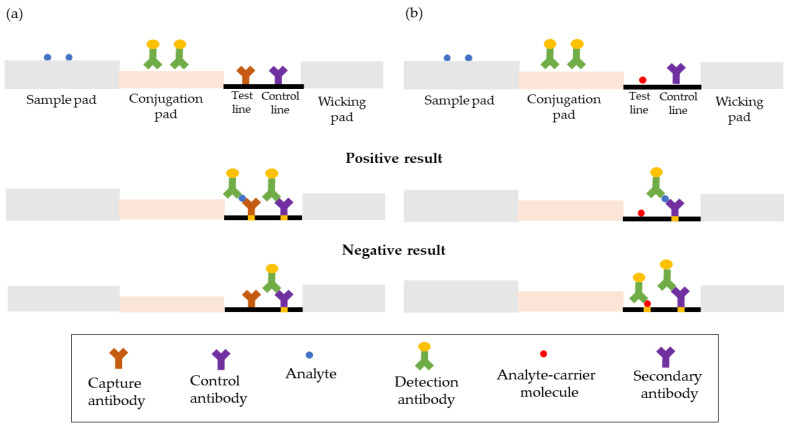
The schematic representation of sandwich (**a**) and competitive (**b**) format of LFA. The capture antibody and control antibody will be lined on the membrane pad, while the detection antibody will be immobilized on the conjugation pad. The sample with the target analyte will be applied on the sample pad and will flow under capillary force to bind with the detection antibody on the membrane pad. In the sandwich format, the color appearing on both lines of the membrane pad will indicate a positive result and if the color only appears on the control line it indicates a negative result. As in a competitive format, the analyte-carrier molecule and secondary antibody will be lined at the test line and control lines on the membrane pad, respectively. The sample with the target analyte will flow to the membrane pad, the presence of color on the control line will indicate a positive result while the presence of color on both lines indicates a negative result.

Typically, the assay format of LFA can be either sandwich or competitive (Figure 3) and should be able to accommodate qualitative, semi-quantitative, and, in limited cases, fully quantitative determinations. Sandwich assay is frequently used to test for bigger analytes having many antigenic sites, such as hCG, dengue antigen, or human immunodeficiency virus (HIV) [60,61,62]. In this case, a positive result is indicated by the presence of a test line. Less than an excess of sample analyte is desired, so that some of the conjugated probes will not be captured at the test line and will continue to flow toward the second line of immobilized antibodies, the control line. This control line typically comprises a species-specific anti-immunoglobulin antibody, specific to the antibody in the conjugated probe. In contrast, a competitive format is employed to screen for tiny compounds with a single antigenic determinant that is unable to bind to two antibodies at once. The absence of a test line on the reaction matrix in this style denotes a successful outcome. Regardless of what happens on the test line, a control line should still form.

### 3.1. The Membrane as an Analytical Region

The type of polymer employed in the membrane, the pore size, quantity, and manner of surfactant administration are often the parameters that determine the performance of the membrane [63]. Although pore size is not an accurate description in the case of nitrocellulose membranes, it ranges from a notional 8 to 15 microns for the membrane used in lateral flow immunoassays. Instead of creating pores, the polymeric structure acts more like a convoluted sponge, providing a channel for the passage of fluids and particles. In comparison to pore size, “wicking rate” or “capillary rise time” are better indicators of membrane flow properties. Capillary rise time, a requirement set by the manufacturer for nitrocellulose membranes, is the period needed for a fluid front to pass through a membrane with a 40 mm width. The kinetics and rate of assay development are crucial, and the selected wicking rate will have a significant impact on the sensitivity and effectiveness of the test [64,65].

Currently, polyethersulfone, nylon, polyethylene, nitrocellulose, and fused silica are utilized to form membrane strips. Due to their inability to bind covalently or in a specific orientation to nitrocellulose, most proteins are known to lose a significant amount of their immunological activity after adhering passively to the membrane surface. Proteins are initially drawn to the membrane surface by electrostatic attraction before attaching to nitrocellulose. Hydrophobic and hydrogen bonds are then used in conjunction to form long-lasting bonds. When designing assays and handling nitrocellulose membranes, it is important to consider the many variables that influence the binding process. Zhu and colleagues demonstrated that the Millipore 180 nitrocellulose membrane was ideal for the detection of soybean mosaic virus (SMV), as it increased the sensitivity and performance compared to Whatman, Millipore 120 and Millipore 135 nitrocellulose membranes. Vivid 90 nitrocellulose membrane from Pall Corporation was used during the construction of the LFA strip for the detection of influenza A and B [66,67].

### 3.2. The Sample Pad

The LFA concept allows for single-step assays with various sample types in various applications. The sample application pad plays a crucial role in making the samples compatible with the assay by treating the sample and releasing the analyte efficiently. The sample pad is usually composed of cellulose or cross-linked silica and must be pre-treated via immersion and drying to avoid introducing sources of variation. The sample pad must accept all the sample volume in a controlled manner, channel fluids into the assay materials, and be treated with assay buffer and other components [66]. The proper preparation of the sample pad material with assay buffer and other essential components, followed by careful drying before use, is crucial for LFA to be efficient. A controlled sample volume must be applied to the sample pad material to transport fluids efficiently to the assay components without causing an overflow [68].

### 3.3. The Conjugate Pad

In close contact with the strip material and the sample application pad is the conjugate release pad, composed of cross-linked silica. The conjugate pad’s glass fiber material has several advantageous properties, such as the ability to hold large volumes, a low level of non-specific binding, and the capability of reliably delivering detector particles onto the membrane within a predetermined volume of sample on the test strip. Owing to these qualities, glass fiber is the best material to utilize in conjugate pads for LFA. Large liquid holding capacities of the conjugate pad allow for effective delivery of the detector particles to the membrane, resulting in extremely sensitive and dependable analyte detection. False positives are less likely since only the target molecule binds to the detector particles due to the low non-specific binding of the glass fiber material [69]. Furthermore, the consistent delivery of the detector particles onto the membrane in a precise volume of the sample allows for reproducible and accurate results, making the glass fiber material an optimal choice for LFA conjugate pads. In LFA, the conjugate pad is pre-treated to improve the release and durability of the labels, which are typically produced from monodisperse latex or AuNP with covalently or passively linked visible or fluorescent dyes. The conjugate release must be effective and consistent throughout the pad’s shelf life, and it is critical to minimize release variance throughout assay tuning [70].

### 3.4. The Wicking Pad

The wick is an essential component of the LFA strip. During the test, it serves as the focal point where the fluid is absorbed and remains attached to the surface. The wick must prevent any fluid from entering the assay again because this could produce inaccurate results. Commonly, the wicking substance is composed of high-density cellulose. The absorbent capacity, affordability, and thickness of the wicking material are only a few of the considerations. While selecting a wicking material for the LFA strip, the roll stock’s availability and tensile strength are also crucial aspects to consider.

### 3.5. The Backing Pad

In LFA, the backing material typically offers a pressure-sensitive adhesive to hold the different parts of the strip in place. The purpose of the backing is to laminate many materials into a single, multipurpose piece. For conventional, non-reader-based lateral flow assays, the lamination technique is crucial because it allows for some tolerance in component overlaps and line positioning in a cassette. The quality of the strip run can be impacted by differences in overlaps, which may be acceptable in some applications, but not in others. This is particularly the case in reader-based systems where the evenness of line development, the speed of running, and the positioning of the fluid front can all significantly affect the performance of the assay [71].

## 4. Commercial LFA for the HBV Detection

According to WHO guidelines, the diagnostic sensitivity and specificity of HBsAg LFA are >99% and >98%, respectively [72]. In February 2019, Abbott announced they had achieved the CE Mark for the world’s most sensitive rapid diagnostic test for the detection of HBsAg named Determine^TM^ HbsAg 2 test for use with serum, plasma or white blood (venous or fingerstick), producing a result in 15 min. The latest update by the WHO on February 2023 is that two HBsAg LFA have been validated (prequalified criteria) and approved by the WHO, including the Determine^TM^ HBsAg 2 [73] and BIOLINE HBsAg WB [74]. This LFA satisfies the regulatory standards of the European Union (EU)’s analytical sensitivity of HBsAg, detecting the 0.13 IU/mL International HBsAg Standard [75,76]. The performance of these LFA is shown in Table 2.

Avellon et al. [77] conducted a multicentre study with the main goal of determining the clinical test performance (sensitivity and specificity) of the Determine^TM^ HBsAg 2 test in serum, plasma, and whole blood samples obtained by venipuncture (all sample types) and fingerstick (whole blood only). The study was conducted on 365 subjects (median age: 49 years) across five clinical sites in the UK and one clinical site in Spain, including a hepatology clinic, gastroenterology clinic, sexual health clinic, digestive clinic and infectious disease clinic. The reference method for the study was performed using a CMIA. Eleven subjects had inappropriate sample processing or no accessible reference results and three participants showed nonreactive results by Elecsys HBsAg II (HbsAg results: 0.07–0.15 IU/mL), but a reactive HBV core antibody result was excluded from the study analyses. Of 351 evaluable subjects, three fingerstick test results had contaminated chase buffer, so were excluded, and one subject had no plasma. Of the total participants, 145 tested positive for HBsAg, while 206 participants tested negative. The results show that the diagnostic sensitivity for venous whole blood, serum and plasma was 92.7%, 97.9% and 98.6%, respectively (at 15 min readings), with 99% to 99.5% specificity. The complexity of the whole blood matrix in comparison to plasma or serum may be the cause of the observed results differing depending on the type of sample used. The ability of Determine^TM^ HBsAg 2 results to be interpreted in 15 min provides a prompt and accurate diagnosis at the point of care [77]. In Ivory Coast, Dembele et al. reported a cross-sectional study phase I from September 2018 to January 2019 carried out in conjunction with the National Programme against viral hepatitis at the National Blood Transfusion Centre (NBTC), the Centre for Diagnosis and Research on AIDS, and the Institut Pasteur of Ivory Coast (IPCI) [78]. In the study, 699 serum and plasma samples were obtained from the biobank of IPCI and CeDRes, and 405 whole blood samples were obtained from blood donors at the NBTC involving serum/plasma and whole blood samples. The samples were evaluated using four HBsAg LFA: Determine^TM^ HBsAg, SD Bioline HBsAg WB^®^, Standard Q HBsAg^®^ and Vikia HBsAg^®^), and the results were calculated with a 95% confidence interval. Two commercially available ELISA tests were used as references. They discovered that the Determine^TM^ HBsAg and Vikia HBsAg^®^ tests performed technically well with specificities of 100% and sensitivities ranging between 99.17% to 100%. While, the SD Bioline WB kits’ sensitivity and specificity ranged from 95.3 to 99.89% and 99.82% to 100%, respectively. There is no significant difference in the performance test evaluated with whole blood and serum/plasma [78]. A multicenter case-control study by Chevaliez et al. [79] to assess the performance of six LFA was evaluated in two laboratories: the French National Reference Center (NRC) for Viral Hepatitis B, C and Delta (Creteil, France) and the Centre Pasteur of Cameroun (Yaounde, Cameroon) between July 2018 and January 2019. The six LFA consist of SD Bioline HBsAg, Hexagon HBsAg, First Response HBsAg Card Test, HBsAg Card, Toyo HBsAg rapid test and VIKIA HBsAg. These were screened on a total of 501 clinical samples (serum = 251, plasma = 250). However, only 459 samples were tested with the SD Bioline HbsAg test due to an insufficient number of available tests. All six LFA had good clinical sensitivity when compared to EIA in serum or CMIA in plasma (98.3–99.3%), and two LFA surpassed 99%. Both VIKIA HBsAg and First Response HBsAg Card tests fulfill the WHO recommendations acceptance criteria in terms of sensitivity and specificity (sensitivity > 99%, specificity > 98%) for prequalification. Four out of the six LFA, including the SD Bioline HBsAg, Hexagon HBsAg, HBsAg Card, and Toyo HBsAg rapid test, had significantly better sensitivity for samples from Cameroon than for samples from France. Low HBsAg levels, mutations in the MHR (Major Hydrophilic Region), low HBV DNA levels, and particular virus genotypes can all result in false-negative results in HBsAg LFA. Although false negative results were infrequent in the study, those who had false-negative results typically had lower levels of HBsAg or amino acid alterations in the MHR of the S gene than those that had true-positive results [79]. Nevertheless, only serum and plasma samples were examined; it is unknown if capillary whole blood examinations would yield comparable outcomes. In India, Shrivastava and Chaurasia [80] and Prabha et al. [81] conducted a cross-sectional study on 532 samples at a tertiary care center located in central India and on 200 blood samples at a tertiary care center at Coimbatore, Tamil Nadu, India, respectively. In their report, Shrivastava and Chaurasia compared the HBsAg LFA of the Meriscreen HBsAg test with the ELISA test. The sensitivity and specificity of the LFA were 96.8% and 99.7%, respectively, compared to ELISA [80]. In contrast, Prabha and colleagues detected HBsAg using HEPAVIEW and performed a comparison against ELISA. The results show that the sensitivity of the LFA was 83.4% with 100% specificity [81]. Screening of 151 samples from the Hepatology Clinic of Civil Hospital, Sukkur, was conducted by Saboor Soomro et al. [82] from June 2018 to December 2018 using RICT, while PCR was used for confirmation. The results show that, in comparison to PCR, the sensitivity and specificity of the LFA were 91.43% and 98.28%, respectively. There were three false-negative samples (5.6%), 32 true-positive samples (94.1%), 114 (97.4%) true-negative samples and just two (2.5%) false-positive samples [82]. These findings are in agreement with a meta-analysis report on 33 LFA using EIA as a reference from 23 countries with pooled sensitivity and specificity of 90.0% and 99.5%, respectively [23]. In comparison to 4^th^ generation ELISA, a study was conducted by Hayder et al. [83] at the Biochemistry and Serology Laboratory of the Pakistan Medical Research Council, Research Centre, Jinnah Post Graduate Medical Centre, Karachi over six months. A total of 200 samples (100 positive HBsAg and 100 negative HBsAg) were tested against three different LFA which are frequently used in Karachi: Acon, Determine, and Intec. The sample size was based on the prevalence of a previous study conducted in Pakistan, where the prevalence was 2.5% for HBsAg at a 95% confidence interval and 3% absolute precision. The sensitivity of HBsAg for Intec and Determine was 98% and for Acon was 95%. Both Intec and Determine showed similar sensitivity. Intec was found to be a cost-effective option for HbsAg screening due to its lower cost [83]. This study showed that all LFA tests were in comparison with the 4th generation ELISA and can be used for screening cases, especially in rural settings.

A cross-sectional study was conducted by Al-Matary et al. [84] to determine the analytical sensitivity of 400 blood donors and recipients attending the Laboratories of Jiblah University Hospital, Yemen, between 2018 and 2019. In the study, the analytical sensitivities and specificities of four LFA: INTEC, SD, ABON and CLUN, were evaluated and compared using the ELISA technique. They found that with INTEC and CLUN, there were more false-negative than false-positive findings for HBV, while with ABON, there were more false-positive than false-negative results. There was a significant difference in sensitivity between ELISA and all the LFA as the sensitivity for INTEC to HBV was 75% (six out of eight cases), for SD 25% (two out of eight cases), for ABON 62.5% (five out of eight cases) and for CLUN 75% (six out of eight cases), which were lower than ELISA. Based on the findings, they did not recommend LFA for blood donor screening as the sensitivity of the LFA needs to be improved [84]. A comparative assessment based on sensitivity was conducted by Navvabi et al. [85] at Urmia Medical University Hospital on 140 men and 60 women using the LFA Ab Core Cassette compared with PCR. The sensitivity and specificity of the test were 97% and 91%, respectively [85]. Sequence differences of nucleotides or amino acids at the target areas could influence in vitro tests for diagnosis. The prevalent genotypes or strains in each geographical area serve as a key indicator of the variety of sequences. Evaluation of diagnostic kits using samples of endemic strains is crucial because standardization by the use of WHO International Standards (WHO IS) only ensures the results of one genotype. A team of Japanese scientists established the regional reference panel (RRP) that contains all predominant HBV genotypes that emerged in Japan, consisting of 64 HbsAg negative and 80 HBsAg positive plasma specimens provided by the Japanese Red Cross Blood Centre, acquired from blood donors in Japan between 2013 to 2015 [86]. A total of 5 HBV DNA quantification kits, 14 HBsAg detection/quantification kits and 2 LFA kits (Determine HBsAg and Determine^TM^ HBsAg 2) were investigated in this study. HBV DNA was found in the WHO IS at a concentration of 10 IU/mL by all five HBV DNA kits. The sensitivity and specificity to the RRP were, respectively, 98.8% and 96.9%. Regardless of HBV genotype, HBV DNA titers were highly associated among the five kits. The minimum detectable amounts in the WHO IS ranged from 0.01 to 0.1 IU/mL for 12 automated HBsAg kits. WHO IS values greater than or equivalent to 1.0 and 0.1 IU/mL were detected by Determine HBsAg and Determine^TM^ HBsAg 2, respectively, with sensitivities between 93.8% and 100%, as the Determine^TM^ HBsAg 2 tested positive for all positive RRP specimens [86]. When HBsAg kits were analyzed by genotype, there were variances in the correlations of the measurements. Variations in amino acids that rely on a person’s genotype may have an impact on how much HBsAg is present. This research may help in the evaluation of the performance of the in vitro diagnostic kits for HBV infection using the RRP of Japan to ensure the accuracy of the HBV diagnostics. A summary of the commercial LFA for the detection of HBV is shown in Table 3.

Thus, the data obtained from all the studies provide valuable insights for the future enhancement of LFA to improve the rapid screening detection of HBV. Currently, Determine™ HBsAg 2 test kit serves as a benchmark for the improvement of LFA as it shows comparable sensitivity to gold standards of HBV detection.

## 5. Emerging LFA Techniques for HBV Detection

LFA is an effective method for remote or resource-constrained situations as it does not require the need for specialized tools or knowledge. When compared to other laboratory-based methods, LFA’s sensitivity and specificity may be constrained, and it might not be ideal for all uses. For example, the standard LFA for HBsAg detection has a comparatively low limit of detection of 0.7 ng/mL [87]. The false-positive or false-negative results are frequently caused by the sensitivity of the LFA. Factors that may affect the sensitivity include a low level of DNA concentration in comparison to the detection limit, mutations as the antibodies used may not recognize the sequences, a low viral load, and the presence of certain genotypes that reduce the amount of HBsAg. Hence, researchers are exploiting a variety of methods, including probe signal enhancement in serological detection and sample amplification techniques in molecular detection to improve the sensitivity of the LFA. Particularly in areas with limited laboratory resources, the development of LFA for HBV detection has been essential in enabling rapid diagnosis and management of the infection. We will delve deeper into these advancements and explore how they have contributed to the widespread use of LFA for HBV detection.

### 5.1. Probe Signal Enhancement for Serological Detection

Serological detection typically relies on the binding of specific probes to target molecules (antigens or antibodies) present in the sample. The sensitivity of LFA biosensors is significantly influenced by the binding affinity of the accumulated AuNP probe captured on the test line through sandwich-type immunoreactions [88,89,90]. In addition, the diameter of the AuNP also influences the sensitivity of the AuNP-based LFA, as AuNP-sized 20–40 nm has been widely used. The optimization of the AuNP with a narrow size distribution will enhance the sensitivity of LFA. For the detection of HBsAg, Kim et al. [91] fabricated LFA with different sizes of AuNP ranging from 34 nm to 137.8 nm. Based on the minimum decrease of absorbance, the AuNP with the size of 42.7 nm was proposed for high sensitivity detection of HBsAg. The signal visibility of the AuNP-antibody conjugated on LFA is affected by the size of the AuNP, as the intensity of the test line will be reduced with oversized AuNP due to the weakened binding efficiency at the test line. The findings of this study are unsatisfactory as they reported that a higher concentration of antibody (20 µg/mL) is needed to intensify the LFA red color which affects the signal intensity of the LFA. The feasibility study of the developed LFA was not mentioned. In addition, Ji et al. [92] studied the effect of AuNP ranging from 20 nm to 180 nm based on the molar extinction coefficient and affinity properties on the strip. They reported that the 100 nm of AuNP showed a higher molar extinction coefficient and stronger affinity to the target antigen, whereas AuNP with 180 nm showed a lower diffusivity and strong light scattering on the test membrane. The cut-off limit of LFA with AuNP of 100 nm in wet format and dry format was 12.5 and 10 times better than LFA with AuNP of 20 nm (conventional strips). Chen et al. [93] explored the effect of the size of AuNP on the sensitivity of LFIA by assembling the 12 nm hydrophobic AuNP into a large AuNP with a size ranging from 100 nm to 400 nm and determined the sensitivity of the LFA. They found that the limit of detection for LFA with AuNP of 270 nm was 0.46 ng/mL, which was 13.8-fold higher compared with conventional LFA with inter- and intra-assay changed from 79.53% to 110.58% with the CV variation from 2.01% to 13.42%, which showed the acceptable precision of the LFA. Subsequently, Shen et al. [94] demonstrated that the probe signal could be enhanced by dual AuNP by determining the feasibility of the LFA on serum samples. By introducing secondary AuNP binding to the primary AuNP on the test line via the biotin-streptavidin binding, the limit of detection was 0.06 ng/mL, which showed a 30-fold sensitivity increment compared with conventional LFA and was demonstrated with inter- and intra-assays of 6.6% and 8.4%, respectively. A comparison study with ELISA showed that the sensitivity of this assay was comparable.

Semiconductor quantum dots (QD) can be used as a signal amplification probe in LFA for high-sensitivity and quantitative detection. The first QD-based LFIA was developed by Zou et al. [95] to detect a metabolite of chlorpyrifos used in agriculture across the world, with a limit of detection of 1.0 ng/mL. Later, the QD microsphere was developed as fluorescent labels for LFA applications by embedding a large amount of QD in a single polymer or silica microsphere [96,97,98,99]. The evaluation of a QD-based probe was first reported by Shen et al. [100] as they produced as-prepared water dispersible phosphine-free QD for HBsAg detection. The prepared phosphine-free QD demonstrated an efficient marker probe in LFA as the limit of detection reached at least 0.05 ng/mL. Zhang et al. [101] then demonstrated the use of a QD-based probe in the detection of HBsAg using dot-blot immunoassay, and the sensitivity of the test was reported to be 78 pg. Owing to that, Shen et al. explored the use of a QD-based probe in the detection of HBsAg using the LFA platform. The test zone’s fluorescence signal increased as the number of antibodies on the QD-based probe increased, but it decreased after the number of titer-labeled antibodies exceeded the maximum value, which was caused by the variable antibody configuration that degraded the active sites [87]. The developed QD-based LFA had a limit of detection of 75 pg/mL, which was higher than the conventional commercial ELISA method, with a sensitivity of 0.2 ng/mL. To further improve, Rong et al. [102] developed a fluorescent test strip based QD nanobead equipped with a multichannel test cartridge with one shared inlet channel and four fluid delivery channels to detect four infectious disease biomarkers inclusive of HBsAg. The limit of detection of the assay was 0.22 IU/mL with an assay time of 20 min. An et al. [103] successfully synthesized in situ cadmium sulfide nanowires (CdSNW) and the feasibility of the LFA was tested using human spike serum. The response range of the CdSNW-based LFA for HBsAg detection was 0.02–100 ng/mL and the limit of detection was 0.5 ng/mL, which is 10-fold higher sensitivity than the commercial photosensing system.

Another promising alternative reporter for LFA is the magnetic nanoparticle. Europium (Eu) (III) chelate-loaded silica nanoparticles and silica-coated magnetic nanoparticles have been used as a replacement for the gold nanoparticle. Xia et al. [104] reported the use of Eu (III) chelate-loaded silica nanoparticle-based LFA for HBV detection. The developed LFA was tested against 286 clinical serum samples and the results were compared with the ELISA method. The LFA showed a limit of detection of 0.03 µg/L, which was 100 times lower than the colloidal AuNP-based LFA [104]. Meanwhile, Zhang et al. [105] applied silica-coated magnetic nanoparticles to enhance the sensitivity of LFA and tested it with 100 clinical samples. The LFA showed a detection limit of 0.1 pg/mL by the charged-coupled device (CCD) reader and 5 pg/mL by naked eye observation. Cai et al. [106] also constructed LFA by using magnetic nanoparticles for the detection of preS2Ag. The feasibility of the developed LFA was tested against 25 serum samples and ELISA was used for comparison. The quantitative limit of detection of the LFA was 3.6 ng/mL with a sensitivity of 93.3% and specificity of 90%.

Employing a dendritic nanoparticle complex can also improve the sensitivity of LFA. A signal amplification system based on a dendritic nanoparticle complex has been applied for the detection of HBeAg. The formation of dendritic nanoparticle complexes relies on biotinylated-captured monoclonal antibodies, targeting antigen molecules and nanoparticle-labeled detection monoclonal antibodies, which work only in the presence of targeting antigen in the sample solution. The signal amplification based on dendritic nanoparticles conjugated with the LFA system to detect HBeAg was developed by Si et al. [107] and tested against 420 clinical samples (30 serum samples of HBsAg and HBeAg positive, 99 plasma samples of HBsAg and HBeAg positive, and 291 plasma samples of HBsAg negative). The LFA was able to detect HBeAg as low as 9 ng/mL, which was 27-fold sensitive compared with conventional LFA.

One of the earliest serological markers during HBV infection is the antibody to anti-HBc. Anti-HBc measurement plays a pertinent role in the therapeutic management of chronic hepatitis B infection [108,109]. Quantitative anti-HBc can be performed using a double-antigen sandwich ELISA and validated using international anti-HBc standards [48]. Thus, polystyrene Eu (III) chelate microparticle-based LFA was also developed for anti-HBc detection in human serum. The developed assay, when tested on 231 human serum samples, demonstrated a limit of detection of 0.31 IU/mL and exhibited a wide linear range (0.63–6.40 IU/mL) [110].

The conversion of lower energy excitation wavelength into higher energy emission at visible wavelength makes upconverting nanoparticle (UCNP) a reporter free from photobleaching and interference of sample autofluorescence. UCNP offers long-term photostability, sensitivity to various sample detections, and an enhanced signal-to-background noise ratio [111]. Martiskainen et al. [112] developed UCNP reporter based LFA and evaluated the limit of detection of the assay against HBsAg on 100 serum and whole blood with the WHO Third International Standard for HBsAg. The UCNP-LFA has shown a limit of detection of 0.1 IU/mL with a sensitivity of 95.4% (95% CI: 89.5–98.5%) compared with commercial Alere Determine HBsAg LFA with an analytical sensitivity of 3.2 IU/mL with a sensitivity of 87.7% (95% CI: 79.9–93.3%), demonstrating that the developed assay shows 32-fold higher analytical sensitivity. Furthermore, Li et al. [113] developed LFA-based upconverting phosphor technology to detect the HBsAb and tested it on 306 clinical serum samples. The quantitative detection of HBsAb is important for individuals who have received hepatitis B immunoglobulin (HBIg) treatment for preventing maternal and physical transmission. The developed UCNP-LFA has shown high sensitivity (99.18) with a limit of detection of 20 mIU/mL compared to commercial ELISA (97.55%) and EIA Abbott (95.51%). Enhancing the signal from these probes enables better detection and quantification of the target molecules, even at low concentrations. This is especially crucial when dealing with samples that may have low target molecule levels, or when early detection of a specific disease marker is desired. By increasing the signal, probe signal enhancement techniques help improve the reliability and efficiency of serological detection assays, leading to more accurate diagnoses and better patient care.

### 5.2. Sample Amplification Techniques in Molecular Detection

Due to the low viral load frequently present in infected people, sample amplification methods used in molecular detection, such as PCR, are crucial for the identification of HBV. HBV viral load can fluctuate greatly and is frequently too low to be directly identified using standard diagnostic techniques. A nucleic acid-based LFA can be designed for testing the presence of an amplified double-stranded nucleic acid sequence specific to the analyzed organism using primers with two different tags. However, the application of LFA based on DNA as biorecognition is not yet commercially available. Here, we describe several techniques, such as LFA’s architecture modification, isothermal amplification as an alternative to the PCR amplification method, and the utilization of detection labels, that have been used in sample pre-treatment to enhance the detection limit, sensitivity and specificity of LFA-based HBV DNA detection. This amplification step enhances the sensitivity and precision of HBV detection, enabling early identification and infection surveillance. Additionally, amplification methods make it possible to detect HBV even in samples with a low viral load, ensuring that people with early-stage or persistent infections are not overlooked during testing. Overall, sample amplification techniques are essential for improving the sensitivity of HBV detection and patient outcomes through prompt diagnosis and effective infection management.

#### 5.2.1. Architecture Modification

The sensitivity of the LFA can be improved by controlling the flow rate of the liquid. Altering the shape of the pad, the wetting distance of liquid and the pore size of the paper as an irregular-size architecture of the sample pad and a larger strip has improved the sensitivity by 10-fold. In LFA, the reaction time of the fluid has an inverse relationship with the sensitivity of the assay. This means that as the reaction time of the fluid increases, the sensitivity of the LFA decreases. Studies have been carried out by incorporating PDMS–paper [114], a hydrogel–paper hybrid [115], and sponge material into conventional LFA as a shunt. A sponge-shunt-integrated LFA was constructed by Tang et al. [116] to increase the detection limit in the detection of HBV DNA clinical samples. The sponge shunt was added to the LFA between the conjugation pad and nitrocellulose membrane to delay the fluid flow rate. The LFA was tested with 12 clinical blood samples that confirmed HBV infection and the feasibility was compared to unmodified LFA. The unmodified LFA showed a detection limit of 10^4^ copies/mL, while the sponge-shunt-modified LFA showed a lower detection limit of 10^3^ copies/mL [117]. Based on Darcy’s Law for liquid wicking in a sequentially contacted strip, a mathematical model was built using the electrical circuit analogies technique to explain the mechanism of the delaying effect of sponges with different hydrophilic and geometric qualities. Darcy’s Law is a fundamental principle in fluid dynamics that describes the flow of a fluid through a porous medium. It asserts that the permeability and viscosity of the medium, as well as the pressure gradient causing the flow, all affect the rate of fluid flow through a porous medium. Darcy’s Law can be defined mathematically as:

Q = −K (dP/dx) A(1)

where A is the cross-sectional area of the medium, K is its permeability, dP/dx is the pressure gradient driving the flow, and Q is its volumetric flow rate. The equation’s negative sign tells us that flow occurs from high-pressure to low-pressure areas [117].

#### 5.2.2. PCR Replacement Technique

Recombinase polymerase amplification (RPA) is robust, convenient and can be carried out at isothermal temperature. RPA produces a nucleic acid complex that can be amplified using a thermostable polymerase enzyme by employing recombinase enzymes to catalyze strand exchange occurrences. The RPA reaction involves the use of two oligonucleotide primers designed to anneal to specific regions of the target nucleic acid sequence. The primers are complexed with recombinase enzymes, which initiate a strand exchange reaction between the primers and the target sequence. A combination of RPA assay with LFA will greatly improve the point of care test in molecular diagnosis for on-site HBV screening. Rapid molecular detection of HBV DNA using RPA combined with LFIA has been developed with good sensitivity and specificity, as low as 10 copies/reaction and with no cross-reactions with other common pathogens [118]. However, the major limitation of RPA in molecular diagnosis is the nonspecific amplification. Thus, RPA assays can offer an internal quality control measure by including an internal control, guaranteeing that false-negative results brought on by assay failure or inhibition can be recognized. It increases confidence in the precision and dependability of the RPA test by making it easier to discriminate between actual negative results and false negatives brought on by inhibitors or technical problems. To enhance the specificity of the RPA assay in serum samples, betaine was used by Yi et al. [119] as an internal control to alleviate the nonspecific amplification caused by the background DNA. In their study, the plasmid DNA with 680 bp fragment of the s-protein gene was the template for the RPA reaction. The developed LFA was evaluated on 40 donor serum samples (20 HBV positive and 20 HBV negative). Through PAGE analysis, it was confirmed that the nonspecific bands were not observed. It was demonstrated that the addition of 0.8 M betaine to the RPA assay showed 95% consistency compared to qPCR for HBV. The accuracy of betaine-assisted RPA-LFA was reported at 95% for serum sample detection with the detection of 2 copies/µL. The whole process of using betaine-assisted RPA until detection using LFA can be completed in one hour, and hence, could offer an alternative for on-site molecular detection of HBV infection [120]. A semi-quantitative nucleic acid test to detect HBV in perinatal transmission with viral loads greater than 2 × 10^5^ IU/mL was designed by combining RPA with LFA, as reported by Mayran et al. [120]. In their study, a total of 89 plasma samples from blood donors (genotypes A, B, C, D, E and F and one undefined sample) were obtained from the Etablissement Français du Sang (EFS, Saint-Denis, France). Genotype D HBV plasma titrated at the viral load of 1.46 × 10^6^ IU/mL was selected as the HBV standard for the selection of primers and the HBV probe was designed to be compatible with either the RPA exonuclease III (RPA-Exo) or RPA endonuclease IV (RPA-NFO) kits. The samples’ amplification step before the RPA amplification was simplified by reducing the reagent used using Chelex 100 resin rather than commercial kits. This method detected the viral loads at a limit of 1.17 × 10^4^ IU/mL. To match with the viral load threshold of 2 × 10^5^ IU/mL used for initiation of perinatal transmission, the samples were extracted in a 10-fold higher volume. Under these circumstances, the assay’s analytical performance had a sensitivity of 98% and a specificity of 88% with an accuracy of 0.99. However, the presence of the internal control will not invalidate false-negative HBV samples caused by mutations as it cannot detect specific genetic mutations that lead to false-negative HBV results. The internal control is co-amplified with the target sequence during the RPA response. It increases confidence in the precision and dependability of the RPA test by making it easier to discriminate between actual negative results and false negatives brought on by inhibitors or technical problems. Hence, RPA is compatible with LFA in HBV detection since it provides rapid and isothermal amplification of HBV DNA targets. In comparison to conventional PCR-based techniques, the key advantage of this technique is the speed of the amplification process, allowing for quicker detection. RPA can also be carried out at a constant temperature, which simplifies assay preparation and does not require specialized equipment. Another form of isothermal amplification assay is called the recombinase-aided amplification assay (RAA) and multienzyme isothermal rapid amplification (MIRA). The RAA technology uses the recombinase *UvsX* (*E. coli*), single-stranded DNA-binding protein (SSB), and DNA polymerase, whilst MIRA uses four core proteins, namely, recombinase-Rec A, DNA helicase-gp41, single-stranded binding (SSB) protein, and DNA polymerase-DNA pol I. In addition, the MIRA reaction system contains helicase which could react with SSB to form the D-loop rapidly [121]. Both RAA and MIRA assays can simplify and amplify target nucleic acids at a constant temperature, eliminating the need for a thermal cycling process such as PCR. A dual RAA-based LFA is inclusive of internal-controlled duplex RAA and LFA, as equipment-free visual detecting and MIRA-based LFA were developed to detect HBV DNA [122,123]. Previously, Bai et al. developed a singleplex real-time RAA assay using heat-treated samples without conventional DNA extraction with sensitivity and specificity of 95.7% and 100%, respectively [122]. In the study, they reported their findings on two fields RAA-based LFA consisting of duplex real-time RAA assay and RAA-LFA using heat-treated samples without DNA extraction. The use of an internal-controlled duplex-RAA-based field detection was to avoid false negative results. A total of 157 serum samples were detected by duplex real-time assay and RAA-LFA. Compared to commercial quantitative-PCR (q-PCR), the real-time RAA assay and RAA-LFA show a sensitivity of 97.17% and 95.77%, respectively, and the specificity of both was 100% [122]. For the diluted standards ranging from 10^6^ to 10^2^ IU/mL, both tests produced positive findings. Additionally, both assays successfully recognized samples with viral loads ranging from 3.31 × 10^6^ to 5.94 × 10^8^ IU/mL when examining the diagnostic performance of clinical samples, demonstrating their capacity to identify high titers. The increase in the sensitivity of the real-time RAA compared to the first reported assay might be due to the overlap of forward primer with 29 bp sequences with the probe for the HBV target gene to minimize the presence of secondary structure. To prevent false-negative results, competitive internal control must be used. Thus, at the optimal concentration of the internal control plasmid (100 copies), the target with low concentration could be detected. The study shows that RAA is compatible with LFA, the same as RPA, and it provides rapid amplification at a steady temperature. Its applicability for LFA in HBV detection is further enhanced by its simplicity and usability. High sensitivity and specificity are shown by RAA, giving reliable and precise findings. MIRA, on the other hand, allows for the simultaneous detection and measurement of several DNA targets, offering a thorough evaluation of HBV in a single LFA. To identify various HBV strains or variants in a single experiment, this multiplexing feature is especially useful. It speeds up the detection process and increases diagnostic precision. Sun et al. reported a study on a combination of MIRA-based LFA for HBV detection in 45 blood samples with a detection sensitivity of 10 pg and 100% accuracy [123]. In the study, the plasmid DNA with 3418 bp DNA fragment from HBV genotype B was used as a template for the MIRA reaction. The MIRA-LFA gave a limit of detection of 100 fg, while MIRA-gel electrophoresis could be detected from 25 ng to 25 pg. However, the extraction process remains a limitation in the study as they did not combine the DNA extraction step with the rapid amplification. Detecting HBV with mutations in the primer recognition sequence is challenging as there is only one MIRA reaction per LFA strip. The multiplexing capability of MIRA simplifies the detection procedure by allowing the identification of various HBV strains or variations in a single experiment. In addition, MIRA has great sensitivity, enabling it to identify even minute amounts of HBV DNA in samples with low viral loads. Furthermore, MIRA makes it possible to quantify viral DNA, which offers important details on viral load and illness development.

**Figure 4:**
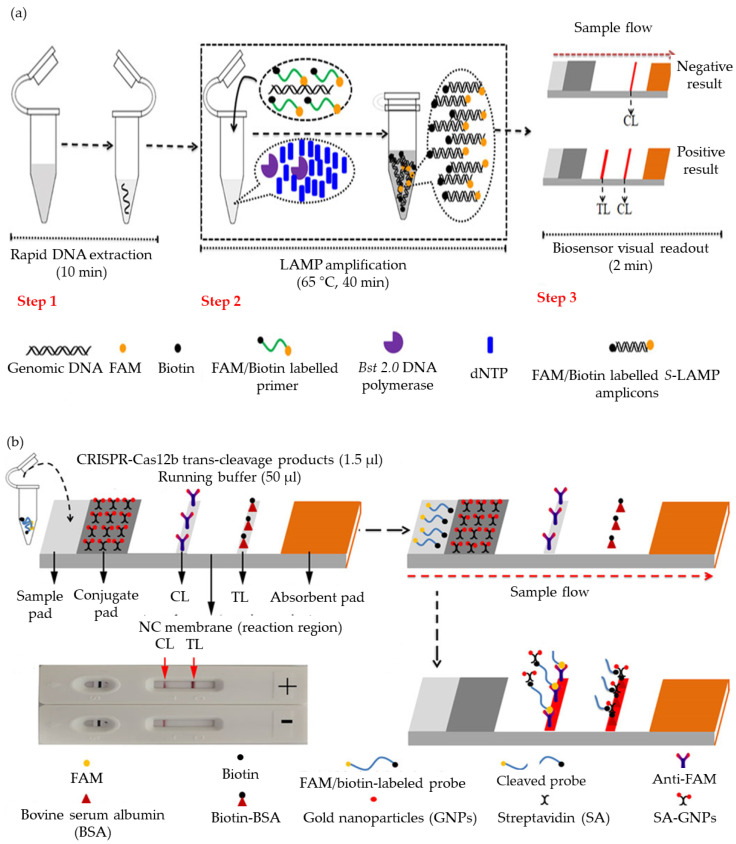
The LAMP-based LFA for HBV detection is shown in (**a**), and the schematic of the visualization of LAMP-LFA-based gold nanoparticles for HBV detection is in (**b**). The process starts with the addition of a running buffer, which moves along the LFA by capillary action. It involves the streptavidin–biotin reaction and the interpretation of the negative and positive samples. “Reprinted with permission from Ref. [126]. © 2021, Chen, Wang, Tan, Huang, Yang and Li”.

Loop-mediated isothermal amplification (LAMP) is used to isothermally amplify a target sequence before ambient temperature. This technique utilizes the big fragment of *Bacillus stearothermophilus* (*Bst*) DNA polymerase in autocycling strand displacement DNA synthesis. The target sequence is amplified for 30 to 60 min at a temperature of approximately 60 °C [124]. Nyan et al. [125] report the utilization of LAMP in the detection of HBV DNA genotypes A, B, C, D, E and F. They used two key components, the Mannitol–Acetate Buffer and *Bst* DNA-polymerase, to facilitate the preparation of the reaction mixture at room temperature and amplify DNA under isothermal conditions while maintaining high sensitivity. The use of a thermostable reaction buffer is crucial because it enables the preparation of the reaction mixture at room temperature, which is more practical and economical than the traditional method of high temperature preparation, which necessitates specialized equipment. The Mannitol–Acetate Buffer can also improve the stability of the reaction mixture, lowering the possibility of DNA templates and amplification products degrading throughout the amplification process. Thus, the use of the Mannitol–Acetate Buffer and *Bst* DNA-polymerase in the above study represents a significant advancement in the field of DNA amplification. The test on 5 µL of LAMP-HBV products using agarose gel stain displayed sensitivities of 10–100 IU per reaction, and a clinical evaluation of 182 blood donor samples (75 positive HBV) showed that the assay could detect all the major genotypes with sensitivity and specificity of 92% and 100%, respectively, with undetected DNA levels below detection limit of ~7–10 IU/rxn [125]. Chen et al. [126] explored the integration of LAMP-based AuNP-LFA to detect HBV DNA; the schematic LAMP detection is shown in Figure 4. The target primers were based on the S gene, and 38 clinical samples (13 HBV positive and 25 non-HBV) were used for analytical sensitivity analysis and confirmed by gel electrophoresis, colorimetric indicator, and real-time turbidimeter which confirmed that the developed LFA was sensitive. The feasibility of the HBV LAMP-LFA tested on 115 serum samples (76 positive HBV by qPCR for sample >30 IU) showed that 78 samples tested positive, indicating the sensitivity of the LAMP-LFA over qPCR. The study concluded that a specific set of LAMP primers based on the S gene was effective in establishing the HBV-LAMP-based LFA assay, which can be carried out at a constant temperature of 65 °C for 40 min. The entire detection process, including HBV DNA preparation, LAMP, and LFA reading, can be completed within 60 min. The HBV-LAMP-based LFA assay had a limit of detection of 7.5 IU per test and a specificity of 100% with no cross-reactivity with other pathogens [126]. Thus, LAMP is an effective and facile isothermal amplification technique with numerous advantages for HBV detection. Even in challenging samples, LAMP has high sensitivity and can find extremely low amounts of HBV DNA. Faster detection times are made possible by rapid amplification of the target DNA sequences, which is advantageous for prompt diagnosis. Additionally, LAMP can be used in conjunction with DNA-intercalating dyes or colorimetric indicators to detect HBV amplification visually without the use of complex equipment.

With better sensitivity and specificity, multiple cross displacement amplification (MCDA) is a novel amplification based on an isothermal strand-displacement polymerization reaction that has been used to detect pathogens, such as mycobacterium tuberculosis, SARS-CoV-2, *Listeria monocytogenes* and *Neisseria meningitides* [127,128,129]. Theoretically, MCDA assay is a diagnostic technique that relies on the use of a polymer with strand displacement activity to amplify a specific target. The established MCDA assay was at least 160 times more sensitive than that of PCR, which has been reported for the detection of *L. monocytogenes*, and it could detect as low as 62.5 fg DNA per reaction [130,131]. Results could be obtained in as little as 15 min using MCDA, which is 16 times more sensitive than LAMP assays. MCDA also offers greater analytical sensitivity than PCR. The low sensitivity of PCR may be attributed to several factors, including the presence of non-target DNA in samples, some PCR-specific inhibitors (such as serum, blood, and food additives), or copy numbers of the target templates that are below the limit of detection. The MCDA approach appears to be more resistant to PCR inhibitors in samples and is unaffected by the presence of non-target sequences [132,133,134]. In their work, Chen et al. [135] developed MCDA-based LFA using primer based on the S gene amplified at 63 °C for one hour and validated for feasibility application on 136 clinical serum samples simultaneously with qPCR. They found that the MCDA-LFA was more sensitive and specific than qPCR, with a limit of detection of 5 IU compared to <30 IU of qPCR. Furthermore, the developed test can be completed within 80 min compared to qPCR which requires 120 to 180 min [135].

The rapid advancement of isothermal amplification has further contributed to the development of isothermal polymerase spiral reaction (PSR). PSR is a rapid and sensitive technique that can amplify DNA or RNA targets in a matter of minutes at a constant temperature, typically 60–65 °C without the need for sophisticated equipment, as for LAMP and MCDA. In contrast, PSR uses a single-stranded DNA template annealed with two primers that contain complementary sequences to the target DNA. The primers are designed to form a spiral structure by complementary base pairing, which facilitates the initiation of polymerization by a DNA polymerase enzyme. A PSR assay containing FITC-labeled DNA probes coupled with LFA was developed by Lin et al. [136] to detect HBV DNA amplified products in 82 plasma samples (29 HBsAg positive-17 genotype B, 9 genotype C, and 3 genotype D). They determined the analytical specificity and sensitivity using PSR-LFA, LAMP and qPCR and performed a sensitivities comparison using ELISA. Under optimized conditions, the sensitivity of the PSR-LFA was found to be 5.4 copies/mL of HBV genomic DNA, which is equivalent to 1.0 IU/reaction. This result was ten times more sensitive compared to qPCR and LAMP techniques, as the detection limit was found to be 5.4 × 10^1^ copies/mL. With 100% specificity consistent with LAMP and qPCR, the PSR-LFA showed no cross-contamination and no false negative or false positive. The feasibility of the PSR-LFA on blood showed 29 HBV positives, which is in agreement with ELISA, while LAMP and qPCR showed 26 positives (89.66%) and 28 positives (96.55), respectively [136].

**Figure 5:**
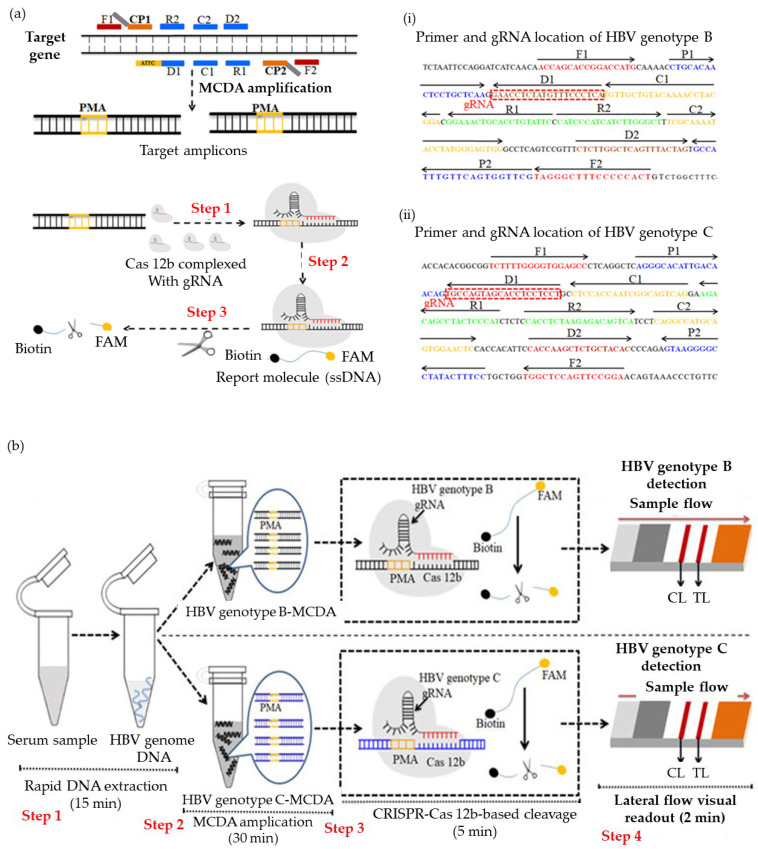
The schematic principle of CRISPR-HBV and the workflow of CRISPR-HBV-LFA systems. The principle of MCDA with modified primer (A), CRISPR-Cas detection (B) and information on the S gene of HBV genotypes B and C (C), are shown in (**a**). Meanwhile, the workflow of the CRISPR-HBV-LFA system, consisting of four steps—DNA extraction, MCDA, CRISPR-Cas, and LFA—is depicted in (**b**). “Reprinted with permission from Ref. [137]. © 2021 Chen, Tan, Wang, Wu, Liu, Yang, Wang, Tai and Li”.

Contrary to previous assays, the Clustered Regularly Interspaced Short Palindromic Repeat (CRISPR)/CRISPR-associated protein (Cas) system is a nuclease-based approach that is extremely sensitive and specific. It consists mostly of Cas proteins and CRISPR RNA (crRNA). Since the creation of CRISPR/Cas detection technology, several platforms have been developed and enhanced—including SHERLOCK (Cas13a), DETECTR (Cas12a), CDetection (Cas12b), and Cas14-DETECTR. In the detection of HBV, the CRISPR/Cas system can detect very small amounts of DNA and identify genetic differences in HBV, enabling early diagnosis and treatment. CRISPR/Cas ensures accurate and consistent detection while reducing the occurrence of false positives and cross-reactivity by specifically targeting the HBV DNA sequence. It is especially useful in resource-constrained areas where access to centralized laboratories may be restricted. Thus, Chen et al. [137] developed a CRISPR–based testing platform integrated with preamplification of MCDA, using LFA as a readout for HBV B and HBV-C genotypes detection, and evaluated the feasibility on 114 serum samples. The primers and gRNAs for genotypes B and C based on gene S were designed for MCDA and CRISPR-Cas12b detection. They also added a protospacer adjacent motif (PAM) to each MCDA primer to detect any sequences that meet the primer design for the CRISPR-Cas12b-based assay. The overview of the CRISPR-Cas-HBV-LFA is shown in Figure 5. The assay could be completed within one hour and the tested sensitivity of the CRISPR-HBV was 10 copies/test with 100% specificity, and no cross-reactions were observed with other HBV genotypes and pathogens [137]. However, since CRISPR/Cas detection needs specialized equipment, an alternative assay, a LAMP-Cas12a assay for the detection of HBV DNA was developed. The assay was integrated with LFA strips technology, enabling rapid detection on 73 serum samples with a low detection limit of detection 1 copy/µL HBV DNA, which is higher than qPCR and ELISA [138].

#### 5.2.3. Detection Label

In practical applications, detection labels play a significant role in the overall analytical performance of LFA. Identification labels are stable substances that can undergo a variety of functionalization and conjugation techniques to increase the biocomponent’s affinity parameters during the target analyte’s detection process. The advancements in detection methodology and equipment have coexisted with the creation of labels or markers for LFA.

Electrochemical detection of nucleic acid can be conducted using a signal-off and signal-on approach. In the signal-off approach, the current response of the mediator/reporter decreases upon using DNA hybridization, as the rigidified structure of the DNA duplex hinders electron transfer between the electroactive reporter and the electrode [139]. This approach has limitations in terms of limited signaling capacity and a high background signal. The signal-on approach, on the other hand, is achieved by metallization of the DNA. Electrochemical detection combined with LFA is based on the use of redox tracers which can be quantitively measured by exploiting electron transfer processes that happen at the interface of the dedicated electrodes on the LFA membrane. An electrochemical DNA-based LFA for signal-on detection employing gold metallization detection was constructed by Srisomwat et al. [140]. In the assay, high performance pyrrolidinyl peptide nucleic acid was used as a recognition probe to capture HBV DNA, as the probe offers high sensitivity and can lower the response of the background. The assay was carried out in a single operation of sample loading and only took 7 min to complete the reaction. The electrochemical HBV-DNA-based LFA had a limit of detection of 7.23 pM and linearity between 10 pM and 2 µM [140].

Gong et al. [141] developed a miniaturized UCNP-LFA integrated with a smartphone for analysis of the results. The fluorescent signal emitted by the UCNP on the conjugate membrane of LFA is captured by the camera of the smartphone and converted to sample concentration through the analysis application. One of their target detections was HBV DNA, through the nucleic acid hybridization method. However, the fluorescence intensities of the UCNP-LFA were lower compared to the gold standard method due to the difference in the exposure between the camera and smartphone. Furthermore, they only used spike serum to test for the sensitivity performance of the LFA. The overall limit of detection of this platform was lower by approximately 5–10-fold compared with the clinical cut-off value [141]. This research shows that the UCNP-LFA platform is sensitive and can be used for multiplex detection. A summary of the current LFA technology innovations for hepatitis B detection can be found in Table 4.

## 6. Conclusions and Future Perspectives

Attesting to the official release statement of the WHO in October 2015 on reducing the morbidity and mortality associated with chronic HBV infections by 2030, numerous techniques for the screening and diagnosis of the viral infection have been developed. Owing to the criteria developed by the WHO, LFA shows an attractive alternative test that is rapid, simple, robust, and inexpensive. In our review, there is rapidly growing research on the existing and in-progress LFA, for serological and molecular testing. Numerous LFA for HBV testing have been developed around the world and are being tested for their feasibility in comparison to the gold-standard methods. Scientists are working to improve the sensitivity and performance of the LFA by applying several techniques. In serological detection, the signal of the probe (antibody/antigen) on the LFA is enhanced by varying the particle size of the nanoparticle and the use of fluorescent nanoparticles. However, the study needs to be tested using enough clinical samples diagnosed with HBV infection to obtain accurate and reliable results for real-world application. Having an intense signal probe will result in accurate screening and lessen the false positive or false negative in qualitative reading. False-negative results provide a risk for silent illness transmission and spread among people, and they also spur interest in highly sensitive LFA. For quantitative analysis, studies have been conducted by conjugating either the antibody or antigen’s capturing/detecting probe with labels such as modified nanoparticles and quantum dots. These nanoparticles not only increase the sensitivity and limit of detection but also bring the LFA towards quantitative detection. As for molecular detection, the rapid development of the LFA has taken place through the PCR replacement technique by integrating the HBV DNA amplification technology with LFA. We discussed how current technology, such as RPA, MIRA, LAMP, PSR, MCDA and CRISPR/Cas, can contribute to the high sensitivity and specificity of HBV detection as they are rapid, can run at room temperature/isothermal temperature, and can detect a small volume of sample and samples with low concentrations, as these factors can cause false-positive and false-negative results. The applied methods can also determine the genotype of the HBV clinical samples by functionalizing the LFA with genotype-specific monoclonal antibodies on multiple capture lines in the form of pattern recognition [142,143]. In addition, the modification of the structure of the LFA has also shown remarkable improvement in the limit of detection. Towards commercialization, a feasibility study needs to be carried out on the targeted population consisting of people of all ages who visited participating clinical facilities for HBsAg screening (high-risk population) or routine follow-up of confirmed HBV, as well as pertinent control populations, such as patients with hepatitis A, hepatitis C, and HIV. Multiplex LFA for HBV to detect different serology markers will also lead to effective vaccination. LFAs can ascertain the immunological response following immunization and confirm protective immunity by testing anti-HB antibodies.

At present, the need for an alternative antigen or antibody as probes in LFA, the miniaturization of the LFA and the utilization of artificial intelligence tools are other vital methods to increase the sensitivity of LFA. The use of aptamers, a single-stranded nucleic acid ligand, as an alternative to capture or detection probes is advantageous as aptamers are robust and can overcome the reproducible production of antibodies/antigens. The selection of the aptamers can be performed using an in-vitro technology called systematic evolution of ligands by exponential enrichment (SELEX), which involves the enrichment of aptamers with a strong affinity for the target molecules [144,145]. Research has been carried out to study the usability of aptamers for antibody or antigen detection. For example, Liu et al. reported the selection of RNA aptamers that can bind to HBsAg and HBsAg-positive hepatocytes with specificity [146]; Zhang et al. successfully screened DNA aptamer targeting HBc [147]; Huang et al. published the DNA aptamers-based fluorescence biosensor that specifically binds to HBeAg using HBeAg-modified magnetic beads as the targets [148]; and Xi et al. developed a novel chemiluminescent aptasensor-based on quick magnetic separation and double-functionalized gold nanoparticles using an HBsAg-specific aptamer [149]. By incorporating aptamers as the recognition elements, LFA for HBV detection became more accessible, affordable, and easy to use. Yet, the applicability of aptamers as an alternative to antibodies for HBV detection in LFA technology has not been explored. Microfluidic technology can be a good platform for complex diagnostics tasks, such as sample preparation, amplification and target molecule detection. Small amounts of fluid can be manipulated and controlled using microfluidic technology on a chip-scale platform. This makes it possible to combine numerous tasks, including sample handling, mixing, and detection, within a compact and portable device for efficient sample loading, distribution, and movement through the assay. This is particularly beneficial in resource-limited settings, or when dealing with precious or limited samples, leading to cost savings and increased affordability. Microfluidics can enhance the binding kinetics between the target analyte and the capture probes by carefully manipulating the flow rates and reaction durations, resulting in higher sensitivity and lower detection limits. Additionally, accurate diagnosis of HBV can be achieved by simultaneously detecting all the key biomarkers by employing multiple probe detection. In multiple probe detection, the use of microfluidic technology reduces the volume of the samples and the possibility of cross-contamination during the sample’s preparation and testing. Built-in detection and quantification processes can be incorporated into microfluidic-based LFA, which can produce more precise and dependable quantitative results. Microfluidic platforms can also incorporate data analysis algorithms, enabling automated data processing and results interpretation while reducing subjectivity and human error. By improving the sensitivity, specificity, speed, and accuracy of LFA, these technologies make it possible for more precise disease detection, monitoring and personalized treatment approaches.

In addition, with the WHO targeting the eradication of viral hepatitis by 2030 and the current HCV cure, there will be a strong focus on viral hepatitis screening and diagnosis to tackle these difficulties. We should not pass up the chance to screen for HBV in addition to HCV as we scale up public health initiatives, including testing and treatment techniques. To boost the testing and diagnosis rates, point-of-care tests will be a crucial tool. However, it is crucial that the tests be robust and have high diagnostic sensitivity and specificity.

## Figures and Tables

**Table 1:** HBV biomarkers and their diagnostic roles.

Markers	Clinical Interpretation
HBV DNA	HBV DNA levels are detected 30 days after infection, often peak during acute hepatitis, and then progressively decline and vanish as the illness goes away on its own. HBV DNA can be found roughly 21 days before HBsAg generally manifests in the blood.
HBsAg	Present during acute infection and continues to be detectable during the chronic infection, when the HBsAg remains detectable for greater than six months. If negative, chronic HBV infection is typically ruled out.
Anti-HBs	A protein produced by the body’s immune system in response to the presence of HBsAg. If negative, the patient has no apparent immunity to HBV.
HBcAg	A marker of HBV infection and can be used in diagnostic tests to detect the presence of the virus. It is not infectious on its own and does not cause disease, but it is an indicator of active HBV replication.
Anti-HBc	For acute infection, the IgM subtype of anti-HBc is seen. If negative, past infection with HBV is typically ruled out.
HBeAg	The secretory form of the nucleocapsid of the HBV can be detected in the serum of a patient in the immune tolerance of the reactivation phase. This antigen can be used to track the development of chronic HBV selectively.
HBeAb	An antibody produced by the body’s immune system in response to HBeAg. It is a marker of the resolution of illness. On rare occasions, carriers can show both HBeAg and an anti-HBe.

HBV: hepatitis B virus; DNA: deoxyribonucleic acid; HBsAg: hepatitis B surface antigen; Anti-HBs: hepatitis B surface antibody; HBcAg: hepatitis B core antigen; Anti-HBc: hepatitis B core antibody; HBeAg: hepatitis B envelop antigen; HBeAb: hepatitis B envelop antibody; Anti-HBe: anti-hepatitis Be protein antibody; IgM: immunoglobulin M.

**Table 2:** Performance characteristics of WHO-prequalified HBV LFA.

Product Name	Manufacturer	Volume/Assay	Sensitivity% (95% CI)	Specificity% (95% CI)	Invalid Rate	Inter-Reader Variability
Determine^TM^ HBsAg 2	Abbott Diagnostics Medical Co., Ltd., Chiba, Japan	50 µL	100 (98.2–100)	100 (98.8–100)	0.12%	0
BIOLINE HBsAg WB	Abbott Diagnostics Korea Inc., Giheung-gu, Korea	100 µL	100 (98.1–100)	99.0 (97.2–99.8)	0.2%	0.2%

CI: confidence interval, µL: microliter.

**Table 3:** Commercial LFA tests for the detection of HBV infection.

Study Site	LFA-Tested	Reference Kit	Number of Samples	Sensitivity% (95% CI)	Specificity% (95% CI)
Multiple sites in Europe [77]	Determine^TM^ HBsAg 2	Abbott ARCHITECT quantitative HBsAg (cut-off 0.13 IU/mL)	348 fingerstick whole blood	97.2 (93.1, 99.2) (15 min) 97.2 (93.1, 99.2) (30 min)	100.0 (98.2, 100.0) (15 min) 100.0 (98.2, 100.0) (30 min)
			348 venous whole blood	97.2 (93.1, 99.2) (15 min) 97.2 (93.1, 99.2) (30 min)	100.0 (98.2, 100.0) (15 min) 100.0 (98.2, 100.0) (30 min)
			347 plasma	98.6 (95.1, 99.8) (15 min) 100.0 (97.5, 100.0)	100.0 (98.2, 100.0) (15 min) 100.0 (98.2, 100.0)
			348 serum	97.9 (94.1, 99.6) (15 min) 100.0 (97.5, 100.0)	99.5 (97.3, 100.0) (15 min) 99.5 (97.3, 100.0)
Ivory Coast [78]	Determine™ HBsAg (Alere International Limited, Ballybrit Galway, Ireland)	Dia.Pro HBsAg^®^ one version ULTRA (Diagnostic Bio Probes Srl, Milano, Italy) Monolisa™ HBsAg ULTRA (BIO-RAD, Marnes-la-coquette, France)	699 serum and plasma 405 whole blood	100 (99.17–100)	100 (99.17–100)
	Vikia HBsAg^®^ tests (Biomérieux, Marcy l’étoile, France)			100 (99.17–100)	100 (99.17–100)
	SD Bioline HBsAg WB^®^ (Standard Diagnostics Inc., Korea)			99.46 (98.33–99.89)	99.82 (98.88–100)
	Standard Q HBsAg^®^ (SD Biosensor, India)			97.1 (95.30–98.24)	99.82 (98.88–100)
Creteil, France and Yaounde, Cameroon [79]	SD Bioline HBsAg (Abbott)	Architect automated device (Architext HBsAg Qualitative assay, Abbott Diagnostics, Chicago, IL, USA) Cobas AmpliPrep/Cobas TaqMan HBV test, version 2.0 (CAP/CTM, Roche Molecular Systems, Pleasanton, CA, USA)	209 serum 250 plasma	98.3 (96.0−99.4) ‘Pooled’	99.4 (96.8−100) ‘Pooled’
	Hexagon HBsAg (Human Diagnostics)		251 serum 250 plasma	98.3 (96.2−99.4) ‘Pooled’	99.5 (97.2−100) ‘Pooled’
	First Response HBsAg Card Test (Premier Medical Corporation)			99.0 (97.1−99.8) ‘Pooled’	99.0 (96.4−99.9) ‘Pooled’
	HBsAg Card (Cypress Diagnostics)			98.3 (96.2−99.4) ‘Pooled’	98.0 (94.9−99.4) ‘Pooled’
	Toyo HBsAg rapid test (Turklab)			98.3 (96.2−99.4) ‘Pooled’	99.5 (97.2−100) ‘Pooled’
	VIKIA HBsAg (bioMerieux)			99.3 (97.6−99.9) ‘Pooled’	99.0 (96.4−99.9) ‘Pooled’
Central India [80]	Meriscreen HBsAg test (Meril Diagnostics)	SD HBsAg ELISA 3.0 (SD Biostandard Diagnostics Private Ltd., Haryana, India)	526 samples	96.8	99.7
Coimbatore, Tamil Nadu, India [81]	HEPAVIEW (Viola Diagnostic System, Diagnostics Pvt., Ltd.)	Merilisa HBsAg (Meril Diagnostics Pvt., Ltd., Gujarat, India)	200 serum	83.4	100.0
Sukkur, Pakistan [82]	Determine (Abbott, Chicago, IL, USA)	PCR (Macrogen, Seoul, Korea)	151 samples	91.43	98.28
Karachi, Pakistan [83]	Acon USA	4th generation ELISA	400 samples	95.0	100.0
	Intec Chin			98.0	100.0
	Determine Abbot			98.0	100.0
Yemen [84]	INTEC	ELISA	400 blood	75.0	98.0
	SD			25.0	98.5
	ABON			62.5	99.7
	CLUN			75.0	95.9
Iran [85]	Ab Core Cassette (Rojan Azma Company)	Gene kit (HBV RQ Ref. #HBV0913A- V3.2)	200 samples	97.0	91.0
Japan [86]	Determine HBsAg (Abbott Diagnostics Medical Co., Tokyo, Japan)	4th WHO IS for HBV DNA (NIBSC code: 10/266)	144 plasma samples	1 IU/mL	93.8
	Determine^TM^ HBsAg 2 (Abbott Diagnostics Medical Co., Chiba, Japan)			0.1 IU/mL	100.0

IU: international unit; min: minute; mL: mililitre; CI: confidence interval.

**Table 4:** Summary of current LFA technology innovations for hepatitis B detection.

Applied Technology	Biomarker	Modification	Sensitivity	Limit of Detection	Number of Samples
Tagged Probe [90]	HBsAg	Size of AuNP	N	20 µg/mL	N
Tagged Probe [92]	HBsAg	Size of AuNP	13.8-fold high	0.46 ng/mL	N
Tagged Probe [93]	HBsAg	Dual AuNP	30-fold high	0.06 ng/mL	N
Tagged Probe [100]	HBsAg	QD	30-fold high	0.06 ng/mL	N
Tagged Probe [101]	HBsAg	QD beads	10-fold high	75 pg/mL	96 serum
Tagged Probe [102]	HBsAg	CdSe/ZnS QD nanobeads	9.09 times	0.22 IU/mL	N
Tagged Probe [103]	HBsAg	CdS nanowires	10-fold high	0.5 ng/mL	N
Tagged Probe [104]	HBsAg	Eu chelate-loaded SiNP	100 times	0.03 µg/L	286 serum
Tagged Probe [105]	HBsAg	SiO2/Fe3O4 nanocomposite	100%	0.1 pg/mL	100 serum
Tagged Probe [106]	preS2Ag	Magnetic NP	93.3%	3.6 ng/mL	25 serum
Tagged Probe [107]	HBeAg	40 nm AuNP	27-fold high	9 ng/mL	420 serum and plasma
Tagged Probe [110]	Anti-HBc	Eu (III) Chelate microparticle	95.9%	0.31 IU/mL	231 serum
Tagged Probe [113]	HBsAb	UCNP phosphor	99.19%	20 mIU/mL	306 serum
RPA [118]	DNA	Isothermal amplification	100%	10 copies/reaction	85 serum
RPA [119]	DNA	Betaine-assisted RPA	90%	20 copies/µL	40 serum
RPA [120]	DNA	DNA extraction	98%	1.17 × 10^4^ IU/mL	89 plasma
MIRA [123]	X and S gene	Isothermal amplification	10 pg	100 fg	45 blood
RAA [122]	DNA	Isothermal amplification	95.7%	1.8 × 10 IU/mL	157 serum
LAMP [125]	DNA	Isothermal amplification	10 IU/reaction	7–10 IU/rxn	182 plasma
LAMP [126]	S gene	LAMP- LFA	10-fold	7.5 IU/test	115 serum
MCDA [135]	S gene	Isothermal amplification	5 IU/reaction	5 IU	136 serum
PSR [136]	S gene	FITC-labeled DNA probe	10 times	5.4 copies/mL	82 plasma
Architecture [117]	DNA	Sponge shunt	10-fold high	10^3^ copies/mL	12 blood
Electrochemical [140]	DNA	Pyrrolidinyl peptide nucleic acid	10 copies/µL	7.23 pM	N
Tagged Probe [141]	DNA	UCNP	10-fold high	0.103 nM	N
CRISPR/Cas [137]	S gene	MCDA & CRISPR/Cas12b	10 copies/ test	1 × 10^8^ copies/µL	114 serum

RPA: recombinase polymerase amplification, LAMP: loop-mediated isothermal amplification, MCDA: multiple cross displacement amplification, RAA: Recombinase-aided amplification, PSR: polymerase spiral reaction, CRISPR/Cas: Clustered regularly interspaced short palindromic repeat (CRISPR)/CRISPR-associated protein (Cas), HBsAg: hepatitis B surface antigen, preS2Ag; pre-S2 antigen, HBeAg: hepatitis B envelop antigen, Anti-HBc; hepatitis B core antibody, HBsAb: hepatitis B surface antibody, DNA; deoxyribonucleic acid, AuNP: gold nanoparticles, QD: quantum dot, UCNP: upconversion nanoparticle, N: not mention, nm; nanometer, pg: picogram, nM: nanomolar, fg: femtogram, ng: nanogram, µL: microlitre, IU: international unit, µg: microgram, NP: nanoparticle.

## Data Availability

Not applicable.
